# Genome sequence of *Lysobacter dokdonensis* DS-58^T^, a gliding bacterium isolated from soil in Dokdo, Korea

**DOI:** 10.1186/s40793-015-0116-8

**Published:** 2015-12-09

**Authors:** Min-Jung Kwak, Soon-Kyeong Kwon, Jung-Hoon Yoon, Jihyun F. Kim

**Affiliations:** Department of Systems Biology and Division of Life Sciences, Yonsei University, 50 Yonsei-ro, Seodaemun-gu, Seoul 120-749 Republic of Korea; Department of Food Science and Biotechnology, Sungkyunkwan University, Suwon, Republic of Korea

**Keywords:** Dokdo, *Xanthomonadaceae*, Protease, Peptidase, Soil bacterium

## Abstract

*Lysobacter dokdonensis* DS-58, belonging to the family *Xanthomonadaceae,* was isolated from a soil sample in Dokdo, Korea in 2011. Strain DS-58 is the type strain of *L. dokdonensis*. In this study, we determined the genome sequence to describe the genomic features including annotation information and COG functional categorization. The draft genome sequence consists of 25 contigs totaling 3,274,406 bp (67.24 % G + C) and contains 3,155 protein coding genes, 2 copies of ribosomal RNA operons, and 48 transfer RNA genes. Among the protein coding genes, 75.91 % of the genes were annotated with a putative function and 87.39 % of the genes were assigned to the COG category. In the genome of *L. dokdonensis*, a large number of genes associated with protein degradation and antibiotic resistance were detected.

## Introduction

The genus *Lysobacter* was firstly described by Christensen and Cook in 1979 as high G + C Gram-negative bacterium with gliding motility [1]. In the past, *Lysobacter* species were classified as “unidentified myxobacters” due to their high G + C ratio and gliding motility. However, the genus *Lysobacter* has features distinctive from myxobacteria and had been proposed as a new genus of *Gammaproteobacteria*. *Lysobacter* species are ubiquitous and have been found in a variety of environments such as soil, water, and the rhizosphere. Currently, more than 30 *Lysobacter* species were registered in the GenBank taxonomy database and among them, 28 species have been validly published [2]. Some of the *Lysobacter* species were known to produce several kinds of lytic enzymes and antibiotics [3] and have an antimicrobial activity against plant pathogens [4]. Moreover, several *Lysobacter* species are known to produce bioactive natural products such as cyclodepsipeptide, cyclic lipodepsipeptide, cephem-type *β*-lactam, and polycyclic tetramate macrolactam [5]. Despite their ubiquitous distribution, many identified species, and possible usefulness as a biocontrol agent, deciphered *Lysobacter* genomes are relatively limited. Here, we present the genome sequence and the genomic information of *Lysobacter dokdonensis* DS-58^T^ (KCTC 12822 ^T^ = DSM1 7958 ^T^), which is the type strain of the species.

## Organism information

### Classification and features

**Fig. 1:**
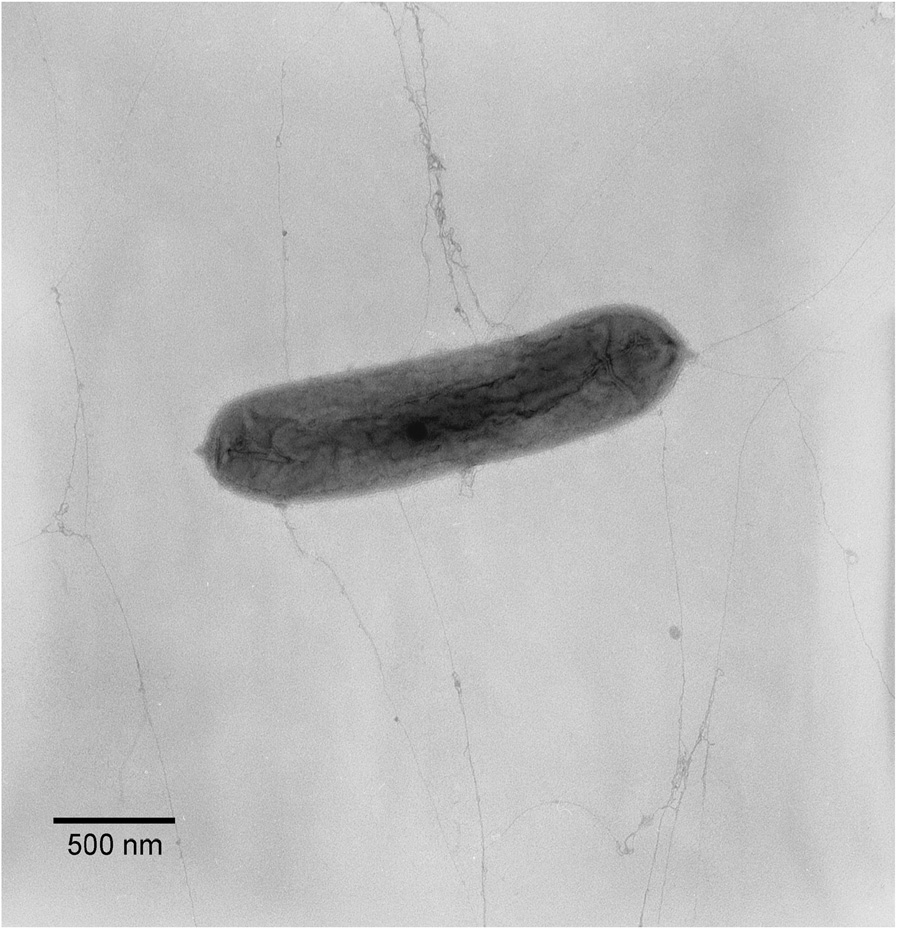
Transmission electron microscopic image of *Lysobacter dokdonensis* DS-58

**Fig. 2:**
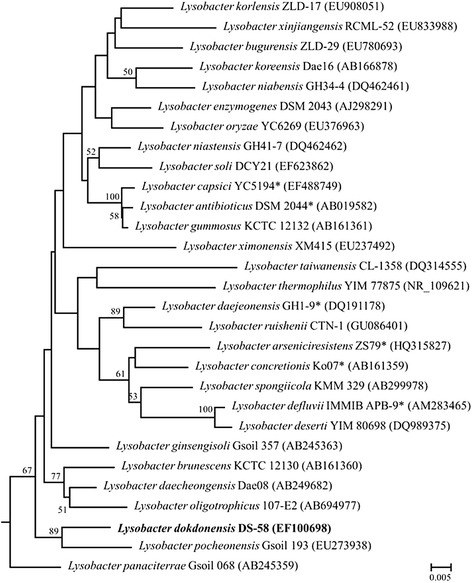
Neighbour-joining tree of the type species of the genus *Lysobacter*. Neighbor-joining tree based on the 16S rRNA gene sequence was constructed using MEGA 5. The evolutionary distances were calculated using Jukes-Cantor model and phylogenetic tree was generated based on the comparison of 1,379 nucleotides. Bootstrap values (percentages of 1,000 replications) greater than 50 % are shown at each node and *Xanthomonas campestris* ATCC 33913 (AE008922) were used as an out-group. The scale bar represents 0.005 nucleotide substitutions per site. Accession numbers of the 16S rRNA gene are presented in the parentheses. *species whose genome has been sequenced

*L. dokdonensis* DS-58^T^ is a Gram-staining-negative, non-motile, and rod-shaped bacterium and was isolated from the soil sample in Dokdo, an island in the East Sea, Korea, in 2011 [6]. *L. dokdonensis* DS-58 grows at the temperature range of 4 to 38 °C, the pH range of 6.0 to 8.0, and the NaCl concentration of 0 to 0.5 % (w/v) [6]. Colony size of *L. dokdonensis* DS-58 is about 1.0 – 2.0 mm on nutrient agar medium and the cell size is 1.0–5.0 μm long and 0.4–0.8 μm wide [6] (Fig. 1). *L. dokdonensis* DS-58 can assimilate dextrin, Tween 40, maltose, *α*-ketobutyric acid, alaninamide, l-alanine, l-alanyl glycine, and l-glutamic acid as a carbon source [6]. Minimum information about a genome sequence (MIGS) for *L. dokdonensis* DS-58 is described in Table 1. Phylogenetically, *L. dokdonensis* DS-58 belongs to the family *Xanthomonadaceae* of the class *Gammaproteobacteria*, and the 16S rRNA gene showed the highest sequence similarity (96.93 %) with *L. niastensis* GH41-7. However, a phylogenetic tree based on the 16S rRNA gene showed that the strain DS-58 is located in the deep branch of the genus *Lysobacter* (Fig. 2).

**Table 1:** Classification and general features of *Lysobacter dokdonensis* DS-58^T^ according to the MIGS recommendations [24]

MIGS ID	Property	Term	Evidence code^a^
	Classification	Domain *Bacteria*	TAS [25]
		Phylum	TAS [26]
		Class	TAS [27]
		Order	TAS [28]
		Family *Xanthomonadaceae*	TAS [29]
		Genus *Lysobacter*	TAS [30, 31]
		Species *Lysobacter dokdonensis*	TAS [6]
		Strain DS-58	TAS [6]
	Gram stain	Negative	TAS [6]
	Cell shape	Rod	TAS [6]
	Motility	Non-motile	TAS [6]
	Sporulation	Non-sporulating	TAS [6]
	Temperature range	4–38 °C	TAS [6]
	Optimum temperature	30 °C	TAS [6]
	pH range; Optimum	6.0–8.0; Optimum 6.5–7.5	TAS [6]
	Carbon source	Dextrin, Tween40, Maltose, L-Alanine, L-Glutamic acid, α-Ketobutyric acid, Alaninamide, L-Alanyl glycine	TAS [6]
MIGS-6	Habitat	Soil	TAS [6]
MIGS-6.3	Salinity	0–0.5 % NaCl (w/v)	TAS [6]
MIGS-22	Oxygen requirement	Aerobic	TAS [6]
MIGS-15	Biotic relationship	Free-living	TAS [6]
MIGS-14	Pathogenicity	Unknown	NAS
MIGS-4	Geographic location	Republic of Korea	TAS [6]
MIGS-5	Sample collection	2011	TAS [6]
MIGS-4.1	Latitude	Not reported	NAS
MIGS-4.2	Longitude	Not reported	NAS
MIGS-4.4	Altitude	Not reported	NAS

^a^ Evidence codes—*IDA* Inferred from Direct Assay, *TAS* Traceable Author Statement (i.e., a direct report exists in the literature), *NAS* Non-traceable Author Statement (i.e., not directly observed for the living, isolated sample, but based on a generally accepted property for the species, or anecdotal evidence). These evidence codes are from the Gene Ontology project [32]

## Genome sequencing information

### Genome project history

The genome sequencing and analysis of *L. dokdonensis* DS-58 were performed by the Laboratory of Microbial Genomics and Systems/Synthetic Biology at Yonsei University using the next generation sequencing. The genomic information was deposited in the GenBank (Accession number is JRKJ00000000). Summary of the genome project is provided in Table 2.

**Table 2:** Genome sequencing project information

MIGS ID	Property	Term
MIGS-31	Finishing quality	High-quality draft
MIGS-28	Libraries used	A 500-bp paired-end library
MIGS-29	Sequencing platforms	HiSeq2000 of Illumina/Solexa
MIGS-31.2	Fold coverage	753-fold coverage
MIGS-30	Assemblers	CLC Genomics Workbench 5.1
MIGS-32	Gene calling method	Glimmer 3
	Locus Tag	LF41
	Genbank ID	JRKJ00000000
	Genbank Date of Release	November 3, 2014
	GOLD ID	Gi0043381
	BIOPROJECT	PRJNA260566
MIGS-13	Source Material Identifier	DS-58
	Project relevance	Environmental, Soil bacterium

### Growth conditions and genomic DNA preparation

*L. dokdonensis* DS-58 (accession numbers of culture collection: KCTC 12822 = DSM1 7958) was routinely cultured on nutrient medium at 30 °C. Strain DS-58 forms light yellow colored colonies with average 1.0–2.0 mm of diameter in 5 days (Table 1) [6]. For the genome sequencing, single colony of *L. dokdonensis* DS-58 was inoculated in nutrient medium and incubated in the shacking incubator at 30 °C. Genomic DNA was extracted using chemical and enzymatic method as described in Molecular Cloning, A Laboratory Manual [7]. Cell lysis was conducted using sodium dodecyl sulfate and proteinase K. From the cell lysate, genomic DNA was purified using phenol:chloroform, precipitated using isopropanol, and finally eluted into Tris-EDTA buffer.

### Genome sequencing and assembly

For the whole genome shotgun sequencing, a library with 500-bp insert size was prepared and paired-end genome sequencing was performed with HiSeq2000 of the Illumina/Solexa platform (Macrogen, Inc., South Korea). Sequence trimming was conducted using CLC Genomics Workbench 5.1 (CLC bio, Qiagen, Netherlands) with parameters of 0.01 quality score and none of the ambiguous nucleotide. Sequence reads below 60 bp in length were discarded. After trimming, a total of 28,810,330 reads with an average read length of 95.8 bp were generated. *De novo* assembly was performed with CLC Genomics Workbench with parameters of automatic word and bubble size, deletion and insertion cost of 3, mismatch cost of 2, similarity fraction of 1.0, length fraction of 0.5, and minimum contig length of 500 bp. After the *de novo* assembly, scaffolding was performed using SSPACE [8] and automatic gap filling was carried out with IMAGE [9]. Following the automatic gap filling, manual gap filling was conducted using CLC Genomics Workbench with the function of Find Broken Pair Mates in the end of the contigs. Basic information of the genome sequencing project is described in Table 2.

### Genome annotation

Structural gene prediction was conducted using Glimmer 3 [10] in RAST server [11] with automatic fixation of errors and frame shifts. Functional assignment of the predicted protein coding sequences (CDSs) was performed using AutoFact [12] with the results of BLASTP or RPS-BLAST with Uniref100, NR, COG, and Pfam databases. For the accurate annotation, the functional assignment results from the RAST server and BLAST were compared each other. When assignment of the gene function was not the same between the results from RAST and BLAST, an additional BLASTP search was performed with NR database at NCBI and the top-hit result was selected for the annotation.

## Genome properties

**Fig. 3:**
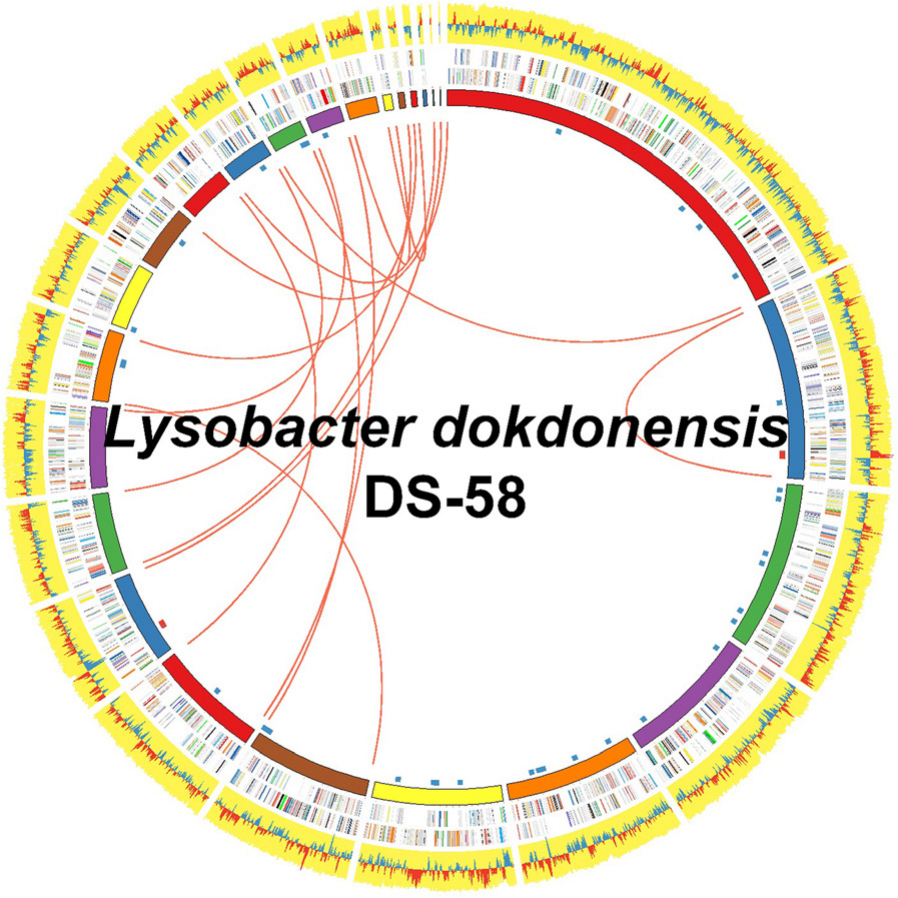
Circular representation of the draft genome of *Lysobacter dokdonensis* DS-58. The first circle from inside shows the 25 contigs sorted by size. The second and the third circles indicate COG- assigned genes in color codes. Yellow circle represents the G + C content and red-blue circle is for the G + C skew. Innermost, blue-scattered spots indicate the tRNA genes and red-scattered spots indicate the rRNA genes. Red lines are to indicate connections of paired-end reads at the end of each contig

The draft genome sequence of the strain DS-58 consists of 25 contigs and the sum of the contigs is 3,274,406 bp (G + C content 67.24 %) (Table 3 and Fig. 3). From the genome of the strain DS-58, 3,155 CDSs, 2 copies of ribosomal RNA operons, and 48 transfer RNAs were detected. Among the predicted CDSs, 2,436 CDSs were annotated with a putative function and 2,757 CDSs were assigned to a COG category. The numbers and percentages of COG assigned genes are shown in Table 4.

**Table 3:** Genome Statistics

Attribute	Value	% of total
Genome size (bp)	3,274,406	100.00
DNA coding (bp)	3,006,255	91.81
DNA G + C (bp)	2,201,865	67.24
DNA contigs	25	-
Total genes	3,209	100.00
Protein coding genes	3,155	98.32
RNA genes	54	1.68
Genes with function prediction	2,436	75.91
Genes assigned to COGs	2,757	85.91
Genes with Pfam domains	2,230	69.49
Genes with signal peptides	456	14.21
Genes with transmembrane helices	767	23.90
CRISPR repeats	1	-

**Table 4:** Number of protein coding genes of *Lysobacter dokdonensis* DS-58 associated with the general COG functional categories

Code	Value	%age^a^	Description
J	168	5.32	Translation, ribosomal structure and biogenesis
A	5	0.16	RNA processing and modification
K	164	5.20	Transcription
L	120	3.80	Replication, recombination and repair
B	1	0.03	Chromatin structure and dynamics
D	34	1.08	Cell cycle control, cell division, chromosome partitioning
Y	0	0.00	Nuclear structure
V	60	1.90	Defense mechanisms
T	232	7.35	Signal transduction mechanisms
M	219	6.94	Cell wall/membrane/envelope biogenesis
N	60	1.90	Cell motility
Z	3	0.10	Cytoskeleton
W	1	0.03	Extracellular structures
U	96	3.04	Intracellular trafficking, secretion, and vesicular transport
O	119	3.77	Posttranslational modification, protein turnover, chaperones
C	142	4.50	Energy production and conversion
G	90	2.85	Carbohydrate transport and metabolism
E	184	5.83	Amino acid transport and metabolism
F	57	1.81	Nucleotide transport and metabolism
H	109	3.45	Coenzyme transport and metabolism
I	114	3.61	Lipid transport and metabolism
P	113	3.58	Inorganic ion transport and metabolism
Q	55	1.74	Secondary metabolites biosynthesis, transport and catabolism
R	308	9.76	General function prediction only
S	303	9.60	Function unknown
-	398	12.61	Not in COGs

^a^The percentages are based on the total number of protein coding genes in the genome

## Insights from the genome sequence

**Fig. 4:**
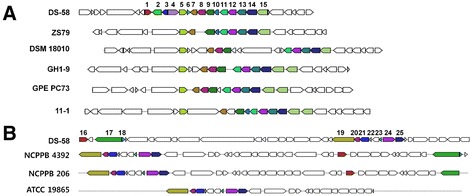
Biosynthetic gene clusters for bacteriocin and arylpolyene. Gene clusters for biosynthesis of secondary metabolites were detected using the AntiSMASH webserver [23]. **a** Bacteriocin-biosynthetic gene cluster. **b** Arylpolyene biosynthetic gene cluster. Same colors in different strains indicate the same genes. White-colored genes are genes unrelated to the secondary metabolite gene clusters. 1, hypothetical protein (LF41_2288); 2, non-heme chloroperoxidase (LF41_2289); 3, alkylhydroperoxidase (LF41_2290); 4, membrane protein-like protein (LF41_2291); 5, 23S rRNA (guanosine-2′-O-)-methyltransferase (LF41_2292); 6, permease (LF41_2293); 7, ribonuclease T (LF41_2294); 8, hypothetical protein (LF41_2295); 9, DUF692 domain containing protein (LF41_2296); 10, hypothetical protein (LF41_2297); 11, phosphate transport system regulatory protein (LF41_2298); 12, phosphate transport ATP-binding protein (LF41_2299); 13, phosphate transport system permease protein (LF41_2300); 14, phosphate transport system permease protein (LF41_2301); 15, phosphate ABC transporter, periplasmic phosphate-binding protein (LF41_2302); 16, coproporphyrinogen-III oxidase (LF41_3101); 17, DNA polymerase I (LF41_3103); 18, DUF2785 domain containing protein (LF41_3104); 19, putative exporter (LF41_3121); 20, fatty acyl-CoA synthetase (LF41_3122); 21, acyltransferase (LF41_3123); 22, dehydratase (LF41_3124); 23, acyl carrier protein (LF41_3126); 24, monooxygenase (LF41_3127); 25, pteridine-dependent deoxygenase (LF41_3128). Strains are: *Lysobacter dokdonensis* DS-58, *Lysobacter arseniciresistens* ZS79, *Arenimonas composti* DSM 18010, *Lysobacter daejeonensis* GH1-9, *Xanthomonas albilineans* GPE PC73, *Pseudoxanthomonas suwonensis* 11–1, *Xanthomonas campestris* NCPPB 4392, *Xanthomonas vasicola* NCPPB 206, *Xanthomonas gardneri* ATCC 19865

Some *Lysobacter* species are known to produce the secondary metabolite with antimicrobial activities [13, 14]. In the genome of *L. dokdonensis* DS-58, biosynthetic gene clusters for a bacteriocin and an arylpolyene were detected. The structure of bacteriocin-biosynthetic gene cluster of DS-58 was similar to the one in *L. arseniciresistens* ZS79 and the structure of arylpolyene-biosynthetic gene cluster was similar to the one in *Xanthomonas campestris*NCPPB 4392 (Fig. 4).

In the genome of *L. dokdonensis* DS-58, a number of genes associated with proteolysis were detected that include 63 genes encoding peptidases and 33 genes encoding proteases. Microbial proteases are among the most important industrial enzymes due to their diverse activities and the genus *Bacillus* is major source of protease in the market [15, 16]. Results from the text mining of annotated gene products indicated that *L. dokdonensis* DS-58 has more genes encoding proteases and peptidases than other genome-sequenced *Lysobacter* species except for *L. antibioticus* ASM73109v1 and *L. capsici* AZ78. Moreover, in the genome of the strain DS-58, genes encoding 17 *β*-lactamases for degrading chemicals such as *β*-lactam antibiotics, biotin-biosynthetic proteins, and type IV fimbrial biogenesis proteins that could be involved in gliding motility were detected.

Distinct from other genera in the *Xanthomonadaceae*, *Lysobacter* spp. exhibit gliding motility [1]. Type IV pili-associated bacterial motility is widespread in members of diverse taxa such as *Proteobacteria*, *Bacteroidetes*, and *Fibrobacteres* [17] and known to be responsible for S-motility in *Myxococcus* and twitching motility in *Lysobacter* [18] as well as *Pseudomonas* and *Neisseria* [19]. Thus, there is a possibility that the gliding motility of *Lysobacter* is associated with type IV fimbriae. On the other hand, GltA, which is involved in A-motility of *Myxococcus xanthus* that best fits the definition of gliding motility [20], was detected in the genome of DS-58 (56 % identity with 88 % coverage).

*Lysobacter* species typically have been isolated from soil and water, but several studies indicated that *Lysobacter* species may survive in more diverse habitats of anaerobic or extreme-cold [21, 22]. A great diversity of secreted degrading enzymes such as proteases and *ß*-lactamases may contribute to the adaptation of *Lysobacter* species to such diverse environments. Abundant genes encoding proteases and peptidases in the genome of DS-58 may contribute to the discovery of effective and commercially useful proteolytic enzymes. Moreover, in the genome of DS-58, dozens of genes involved in the biosynthesis of type IV fimbriae were detected. The mechanism of gliding motility has not yet been clearly revealed, and we expect that the genome information of DS-58 may contribute to the genetic analysis of bacterial gliding motility.

## Conclusions

*L. dokdonensis* DS-58, the type strain of the species, is a soil bacterium isolated from Dokdo in Korea. Through a phylogenetic analysis of the 16S rRNA gene, *L. dokdonensis* is located in a deep branch of the genus *Lysobacter*. The genome sequence of *L. dokdonensis* DS-58 is comprised of 25 contigs of 3,274,406 bp with G + C content of 67.24 %. In the genome of DS-58, a total of 3,155 CDSs were predicted and 87.39 % of the CDSs were functionally assigned to COG categories. Dozens of genes associated with protein degradation and resistance to antibiotics were detected. Through the genome analysis of *L. dokdonensis* DS-58, we report that this soil bacterium harbors a large number of peptidases and proteases, which may represent a rich source of protein-degrading enzymes.

## Abbreviations

COG: Clusters of Orthologous Groups
NR: Non-redundant
Uniref: UniProt Reference Clusters
Pfam: Protein families
SSPACE: SSAKE-based Scaffolding of Pre-Assembled Contigs after Extension
IMAGE: Iterative Mapping and Assembly for Gap Elimination
RAST: Rapid Annotation using Subsystem Technology
AutoFACT: Automatic Functional Annotation and Classification Tool
BLAST: Basic Local Alignment Search Tool
RPS-BLAST: Reversed Position Specific-BLAST
MEGA: Molecular Evolutionary Genetics Analysis
MIGS: Minimum Information about a Genome Sequence
CRISPR: Clustered Regularly Interspaced Short Palindromic Repeat.
